# Characterization of H9N2 Avian Influenza Viruses Isolated from Poultry Products in a Mouse Model

**DOI:** 10.3390/v14040728

**Published:** 2022-03-30

**Authors:** Jurika Murakami, Akihiro Shibata, Gabriele Neumann, Masaki Imai, Tokiko Watanabe, Yoshihiro Kawaoka

**Affiliations:** 1 Division of Virology, Institute of Medical Science, University of Tokyo, Tokyo 108-8639, Japan; murajuri@ims.u-tokyo.ac.jp (J.M.); mimai@ims.u-tokyo.ac.jp (M.I.); 2 Exotic Disease Inspection Division, Laboratory Department, Animal Quarantine Service, Ministry of Agriculture, Forestry and Fisheries, Tokoname 479-0881, Japan; akihiro_shibata280@maff.go.jp; 3 Department of Pathobiological Sciences, School of Veterinary Medicine, University of Wisconsin–Madison, Madison, WI 53706, USA; gabriele.neumann@wisc.edu; 4 The Research Center for Global Viral Diseases, National Center for Global Health and Medicine Research Institute, Tokyo 162-8655, Japan; 5 Department of Molecular Virology, Research Institute for Microbial Diseases, Osaka University, Suita 565-0871, Japan; 6 Center for Infectious Disease and Education and Research (CiDER), Osaka University, Suita 565-0871, Japan

**Keywords:** H9N2, avian influenza viruses, pathogenicity and replicative ability in mammals

## Abstract

Low pathogenic H9N2 avian influenza viruses have spread in wild birds and poultry worldwide. Recently, the number of human cases of H9N2 virus infection has increased in China and other countries, heightening pandemic concerns. In Japan, H9N2 viruses are not yet enzootic; however, avian influenza viruses, including H5N1, H7N9, H5N6, and H9N2, have been repeatedly detected in raw poultry meat carried by international flight passengers from Asian countries to Japan. Although H9N2 virus-contaminated poultry products intercepted by the animal quarantine service at the Japan border have been characterized in chickens and ducks, the biological properties of those H9N2 viruses in mammals remain unclear. Here, we characterized the biological features of two H9N2 virus isolates [A/chicken/Japan/AQ-HE28-50/2016 (Ck/HE28-50) and A/chicken/Japan/AQ-HE28-57/2016 (Ck/HE28-57)] in a mouse model. We found that these H9N2 viruses replicate well in the respiratory tract of infected mice without adaptation, and that Ck/HE28-57 caused body weight loss in the infected mice. Our results indicate that H9N2 avian influenza viruses isolated from raw chicken meat products illegally brought to Japan can potentially infect and cause disease in mammals.

## 1. Introduction

H9N2 avian influenza viruses are of low pathogenicity in chickens but still cause mild respiratory symptoms and a drop in egg production, leading to economic losses in countries that rely on the poultry industry [1]. The H9N2 virus were first isolated in turkeys in the United States in 1966 [2,3]. Since the late 1990s, H9N2 viruses have been widespread in various species of wild birds and domestic poultry across the world, and have occasionally expanded their host range to mammalian species, such as pigs and humans [4]. The first case of human infection with a H9N2 virus was reported in China in 1998 [5]; since then, as of 20 December 2021, a total of 95 laboratory-confirmed cases of human infection with H9N2 viruses have been reported mainly in China, but also in other countries including Egypt, Bangladesh, Cambodia, Oman, Pakistan, India, and Senegal [6,7]. In addition, recent genetic analyses have revealed that H9N2 avian influenza viruses have contributed to the genetic and geographic diversity of H5N1 avian influenza viruses through genetic reassortment [8,9]. Furthermore, novel H7N9 avian influenza viruses, which emerged in 2013 and have infected a substantial number of people in China, are reassortants whose six internal genes come from H9N2 viruses [10]. Other subtypes of avian influenza viruses, such as H5N6, H5N8, and H10N8, can also possess the internal genes of H9N2 viruses due to genetic reassortment [11]. The worldwide distribution of H9N2 viruses with the ability to infect mammals and humans is increasing concern regarding their pandemic potential.

The HA gene of the H9N2 virus are classified into the Eurasian and American lineages. The Eurasian lineage is further classified into several sub-lineages, including A/duck/Hong Kong/Y280/1997 (Y280), A/quail/Hong Kong/G1/1997 (G1), and A/duck/Hong Kong/Y439/1997 (Y439). Y280-like viruses represent the dominant lineage in China and Southeast Asian countries. G1-like viruses have mainly circulated in South China, Central Asia, the Middle East, and North Africa, whereas Y439-like viruses have circulated in South Korea [12,13]. In Japan, H9N2 viruses are not yet enzootic. Therefore, careful surveillance of avian influenza viruses in poultry products carried by international flight passengers from other countries at the border is important to prevent their spread in Japan. Indeed, avian influenza viruses, including H5N1, H7N9, H5N6, and H9N2, have been repeatedly detected in raw poultry meat brought by international flight passengers from Asian countries to Japan [14,15]. Previous reports showed that seven H9N2 avian influenza viruses were isolated from chicken and duck meat products carried by passengers on flights from China and Vietnam; these viruses were classified into the Y280 sub-lineage [14,15]. One of these H9N2 viruses was characterized in avian models and found to be of low pathogenicity in chickens and ducks [14]; however, the biological properties of these H9N2 viruses in mammals remain unknown. Therefore, we here characterized the biological features of two H9N2 viruses [A/chicken/Japan/AQ-HE28-50/2016 (Ck/HE28-50) and A/chicken/Japan/AQ-HE28-57/2016 (Ck/HE28-57)] isolated from chicken meat products transported to Japan by international passengers from Vietnam [14] in a mouse model.

## 2. Materials and Methods

### 2.1. Cells

Madin-Darby canine kidney (MDCK) cells were maintained in minimal essential medium (MEM) containing 5% newborn calf serum, vitamins, essential amino acids, and antibiotics. Human lung adenocarcinoma epithelial A549 cells were grown in Ham’s F-12K containing 10% fetal bovine serum (FBS) with antibiotics. Chicken embryo fibroblast DF-1 cells were grown in Dulbecco’s modified Eagle medium (DMEM) containing 10% fetal bovine serum (FBS) with antibiotics. MDCK and A549 cells were cultured at 37 °C with 5% CO_2_. DF-1 cells were cultured at 39 °C with 5% CO_2_ unless otherwise stated.

### 2.2. Viruses

Two H9N2 viruses [A/chicken/Japan/AQ-HE28-50/2016 (Ck/HE28-50) and A/chicken/Japan/AQ-HE28-57/2016 (Ck/HE28-57)], which were isolated from chicken meat products found in a traveler’s luggage in 2016 as previously described [14], were used in this study. To make viral stocks, Ck/HE28-50 and Ck/HE28-57 were propagated at 37 °C in 10-day-old, specific-pathogen-free embryonated chicken eggs for 48 h. Virus titers were determined by using plaque assays in MDCK cells.

### 2.3. Viral Genome Sequencing

Viral RNA was extracted from the supernatants of stock viruses by using a QIAamp viral RNA Mini Kit (Qiagen, Hilden, Germany). The first-strand cDNA was synthesized using Uni12 primer, Superscript III (Invitrogen, Carlsbad, CA, USA), and universal primers specific for influenza A virus genes [16]. The resulting products were PCR-amplified using Phusion DNA polymerase (NEW ENGLAND BioLabs, Tokyo, Japan) with specific primers for each virus gene. The amplified PCR products were purified and subjected to direct sequencing using the ABI PRISM 310 system.

### 2.4. Viral Replication Assay

Triplicate wells of confluent MDCK and A549 cells were infected with viruses at a multiplicity of infection (MOI) of 0.001 and incubated for 1 h at 37 °C. After the 1 h incubation, the MDCK and A549 cells were incubated in MEM and Ham’s F-12K containing 0.3% bovine serum albumin (BSA) at 37 °C, respectively. Triplicate wells of confluent DF-1 cells were infected with viruses at an MOI of 0.001, incubated for 1 h at 39 °C, and then incubated in DMEM containing 0.3% BSA at 39 °C. For the MDCK and DF-1 cells, aliquots of supernatants were harvested at 12 h post-infection (hpi), 24 hpi, and 48 hpi. For the A549 cells, aliquots of supernatants were harvested at 24-h intervals. Virus titers in the culture supernatants at each time point were determined utilizing plaque assays in MDCK cells.

### 2.5. Mouse Experiments

Female, six-week-old C57BL/6J mice (Japan SLC) were used for these experiments. To observe body weight changes, 5 mice/group for each virus were anesthetized with isoflurane and inoculated intranasally with 10^6^ PFU/mouse in a 50-μL volume. The mice were monitored daily for clinical signs of infection and checked for changes in body weight and mortality for 14 days post-infection (dpi). Mice were euthanized if they lost more than 25% of their initial body weight. The remaining mice were euthanized at 14 dpi. For virus replication assessment, groups (3 mice/group) were infected intranasally with 10^4^ or 10^6^ PFU/mouse in a 50-μL volume. Three mice in each group were euthanized on Days 3 and 6 post-infection. Organs (lungs and nasal turbinates) were collected for virus titration in plaque assays in MDCK cells. The data shown are the mean virus titers ± standard deviations (SD). All experiments with mice were performed in accordance with the University of Tokyo’s Regulations for Animal Care and Use and were approved by the Animal Experiment Committee of the Institute of Medical Science, the University of Tokyo (PA20-6).

### 2.6. Statistical Analysis

For statistical analyses of growth kinetics data, the virus titer values were converted to the log10 scale, and a two-way ANOVA, followed by a Dunnett’s test, was performed. The virus titers of Ck/HE28-50 were compared with those of Ck/HE28-57, and the difference was considered significant for *p* values of <0.05 in GraphPad Prism6.

## 3. Results

### 3.1. Replicative Ability of Two H9N2 Avian Influenza Viruses In Vitro Subsection

Two H9N2 viruses (Ck/HE28-50 and Ck/HE28-57) were previously isolated from chicken meat products transported by international flight passengers from Vietnam to Japan in 2016 [14] and found to be Y280 lineage H9N2 viruses with an HA cleavage site (PSRSSR/GLF) typical of low pathogenic avian influenza viruses. We found that both Ck/HE28-50 and Ck/HE28-57 possess several molecular markers in their proteins that are associated with the adaptation of avian influenza viruses in mammals (i.e., 5 amino acids in PB2, 1 in PB1, 3 in PA, 4 in HA, 3 in M1, and 3 in NS; as shown in Table 1 and Supplementary Table S1) [17,18,19,20,21]. Moreover, Ck/HE28-57 possesses three additional molecular markers with mammalian-like motifs at positions 292 and 340 in PB2 and 160 in HA (Table 1 and Supplementary Table S1). These viruses were propagated in embryonated chicken eggs to produce viral stocks. The stock titers of Ck/HE28-50 and Ck/HE28-57 were 9.64 log_10_ PFU/mL and 9.61 log_10_ PFU/mL, respectively.

**Figure 1:**
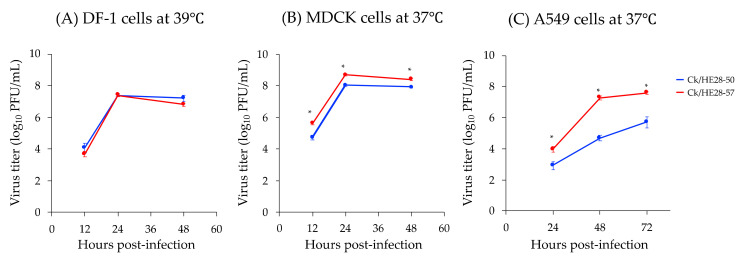
Replicative ability of two H9N2 viruses in DF-1, MDCK, and A549 cells. DF-1 (**A**), MDCK (**B**), and A549 (**C**) cells were infected with Ck/HE28-50 or Ck/HE28-57 at a multiplicity of infection of 0.001 and incubated at the indicated temperatures. Virus titers are presented as the mean ± standard deviation (SD) of two independent experiments. * indicates *p* < 0.05 Ck/HE28-50 versus Ck/HE28-57.

To characterize the replicative ability of the H9N2 viruses in vitro, we examined their growth kinetics in chicken embryo fibroblast DF-1 cells and mammalian (MDCK and A549) cells. In DF-1 cells, Ck/HE28-50 and Ck/HE28-57 both grew well at 39 °C, with peak titers of 7.38 ± 0.09 log_10_ PFU/mL and 7.39 ± 0.09 log_10_ PFU/mL, respectively, at 24 hpi (Figure 1A). In MDCK cells, both viruses replicated well at 37 °C, but the peak titer of Ck/HE28-57 was slightly higher than that of Ck/HE28-50 (i.e., 8.03 ± 0.09 log_10_ PFU/mL and 8.70 ± 0.07 log_10_ PFU/mL at 24 hpi, respectively) (Figure 1B). In contrast, in human lung A549 cells, Ck/HE28-57 replicated more efficiently than Ck/HE28-50 at 37 °C, with a peak titer 2-log higher than that of Ck/HE28-50 (i.e., 7.59 ± 0.08 log_10_ PFU/mL cf. 5.70 ± 0.36 log_10_ PFU/mL) (Figure 1C). These results demonstrate that Ck/HE28-57 replicates more efficiently than Ck/HE28-50 in MDCK and human lung A549 cells, but both viruses grow equally well in DF-1 cells.

### 3.2. Replicative Ability and Pathogenicity of Two H9N2 Avian Influenza Viruses in Mice

**Figure 2:**
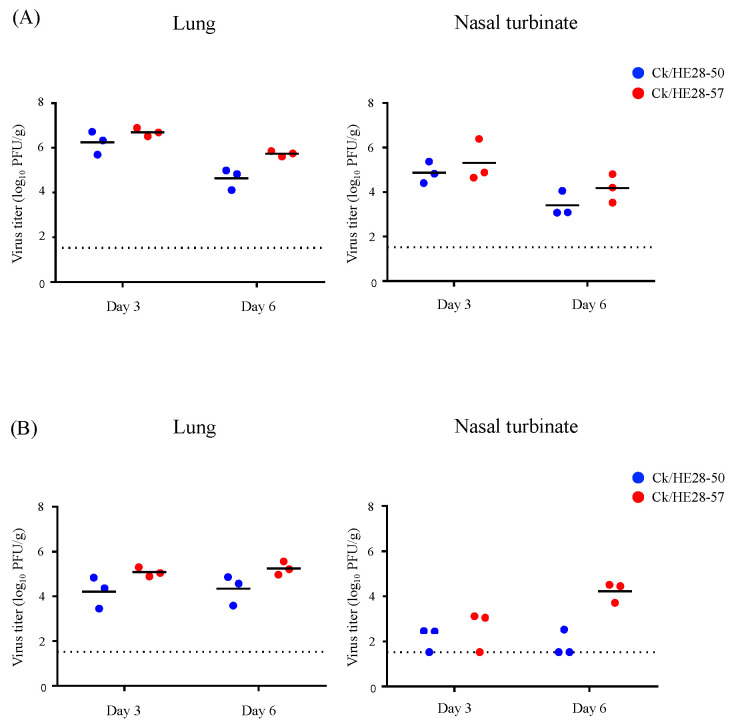
Virus titers in mice infected with H9N2 avian influenza viruses. Three mice per group were intranasally inoculated with (**A**) 10^6^ PFU (in 50 μL) or (**B**) 10^4^ PFU (in 50 μL) of Ck/HE28-50 or Ck/HE28-57. Lung and nasal turbinate tissues were collected from the infected mice at 3 and 6 days post-infection for virus titration. Each circle represents the virus titer of an individual sample. The bar indicates the mean and is shown only when the virus was recovered from all three mice. The lower limit of detection is indicated by the horizontal dashed line.

We next examined the replicative ability of these H9N2 viruses in vivo. C57BL/6 mice were intranasally infected with 10^4^ PFU or 10^6^ PFU of Ck/HE28-50 or Ck/HE28-57, and organ samples were collected at 3 and 6 dpi for virus titration. In mice infected with 10^6^ PFU of the virus, both viruses replicated well in the lungs and nasal turbinates. The mean virus titers in the lungs of mice infected with Ck/HE28-50 or Ck/HE28-57 were 6.25 ± 0.42 and 6.70 ± 0.20 log_10_ PFU/g at 3 dpi, respectively, and those in the nasal turbinates were 4.87 ± 0.40 and 5.31 ± 0.77 log_10_ PFU/g at 3 dpi, respectively, for the Ck/HE28-50 and Ck/HE28-57 groups (Figure 2A). For the mice infected with 10^4^ PFU of the virus, the mean lung titers of the Ck/HE28-50- or Ck/HE28-57-infected mice were 4.32 ± 0.56 and 5.25 ± 0.26 log_10_ PFU/g, respectively, at 6 dpi. In the nasal turbinate, the mean virus titer of Ck/HE28-57-infected mice was 4.22 ± 0.38 log_10_PFU/g at 6 dpi, whereas virus (2.52 log_10_ PFU/g) was recovered from only one of the three mice infected with Ck/HE28-50 (Figure 2B).

**Figure 3:**
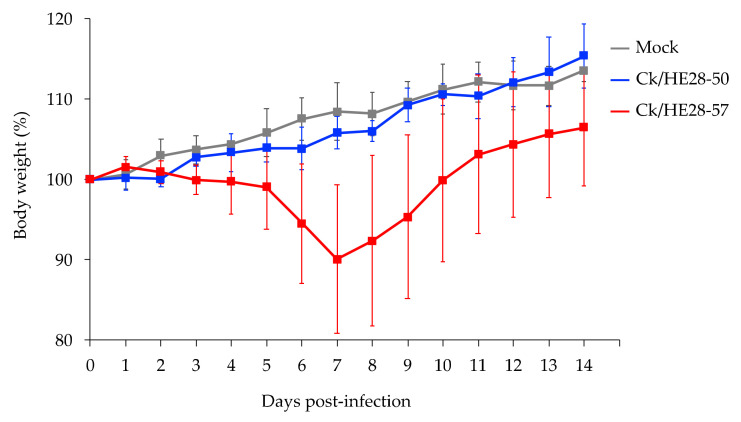
Body weight changes in mice infected with H9N2 avian influenza viruses. Five mice per group were intranasally inoculated with 10^6^ PFU (in 50 μL) of Ck/HE28-50, Ck/HE28-57, or PBS as a control. Body weights were measured daily for 14 days and are presented as means ± SD.

To examine the pathogenicity of the two H9N2 viruses, five C57BL/6 mice per group were inoculated intranasally with 10^6^ PFU of each virus, and their body weight changes were monitored for 14 days (Figure 3). The Ck/HE28-50-infected mice continued to gain body weight during the observation period, as did the control group, which was inoculated intranasally with 50 μL/mouse of PBS. In contrast, the body weight of the Ck/HE28-57-infected mice declined by day 7 post-infection (Figure 3); however, all mice recovered from their infection with Ck/HK28/57.

## 4. Discussion

In this study, we examined the biological features of two H9N2 viruses (Ck/HE28-50 and Ck/HE28-57) that were isolated from raw poultry meat that was illegally brought into Japan from Vietnam [14]. We found that these H9N2 viruses replicate reasonably well in mammalian cells and mice, although the replicative ability of Ck/HE28-50 was lower than that of Ck/HE28-57 in A549 cells (Figure 1). Concerning pathogenicity in mice, Ck/HE28-57 caused a temporary loss of body weight, whereas the body weight of Ck/HE28-50-infected mice gradually increased similarly to the mock-infected group (Figure 3). These data demonstrate that Ck/HE28-50 and Ck/HE28-57 can replicate in mammals and that Ck/HE28-57 is slightly more virulent than Ck/HE28-50 in mammalian hosts.

Avian influenza viruses rarely infect humans due to host range restrictions. Several viral factors determine the viral interspecies transmission and pathogenicity of avian influenza viruses in mammals [17,18,19,20,21]. The receptor-binding specificity of the HA protein is a major determinant of the influenza viral host range [19,22,23,24]. In general, human influenza viruses preferentially bind to sialic acid-α2,6-galactose (SAα2,6Gal), the predominant sialyloligosaccharide species on epithelial cells in the upper respiratory tract of humans [19,25], whereas avian influenza viruses preferentially recognize sialic acid-α2,3-galactose (SAα2,3Gal), the major sialyloligosaccharide species in the duck intestinal tract [26]. A change of binding preference from the avian-type receptor (i.e., SAα2,3Gal) to the human-like receptor (i.e., SAα2,6Gal) is considered to be one of the important steps for the adaptation of avian influenza viruses to mammalian hosts [22,24,25,27]. There are several key residues in HA that affect viral receptor-binding preference; for example, replacing glutamine (Q) with leucine (L) at position 226 (H3 numbering), which is found in most H9N2 poultry isolates, can alter the viral receptor-binding preference from the avian-type receptor to the human-type receptor [23]. Ck/HE28-50 and Ck/HE28-57 possess four amino acid substitutions in HA (i.e., HA-159N, HA-190V, HA-198T, and HA-226L) that are associated with a shift in the binding preference of avian influenza viruses to the human-type receptor (Supplementary Table S1) [23,24,28,29]. In addition, the viral polymerase complex, which comprises PB2, PB1, PA, and NP, has key roles in the adaptation of avian influenza viruses to mammalian hosts, and several amino acid substitutions have been shown to contribute to increased polymerase activity, replicative ability, and/or virulence in mammalian models [20,30,31,32,33,34]. The change from glutamic acid (E) to lysine (K) at position 627 of the PB2 protein is one of the most important host range determinants, but neither Ck/HE28-50 nor Ck/HE28-57 possesses the E-to-K change at position 627 of PB2. Instead, they have several amino acid substitutions in the viral polymerase complex that are associated with the mammalian adaptation and pathogenicity of avian influenza viruses (i.e., PB2-89V and 309D, PB2-504V, PB2-588V, PB1-622G, PA-63I, PA-356R, and PA-383D) (Supplementary Table S1) [20,21,31,33,34,35,36]. Ck/HE28-57, but not Ck/HE28-50, possesses the isoleucine (I)-to-valine (V) mutation at position 292 of PB2 protein that is associated with increased H9N2 virus polymerase activity in mammalian cells and enhanced virulence in mice [37]. In our study, Ck/HE28-57 was more pathogenic than Ck/HE28-50 in mice (Figure 3), indicating that PB2-292V may contribute to the increased virulence of Ck/HE28-57 in the mouse model. Alternatively, an as-of-yet undetermined amino acid change(s) in Ck/HE28-57 may have a role in its pathogenicity in mice. Further studies are needed to clarify the factors that determine the differences between the properties of Ck/HE28-50 and Ck/HE28-57.

Compared with highly pathogenic avian influenza viruses, such as H5N1 and H7N9 viruses, less attention is paid to low pathogenic avian influenza viruses. However, many reports, including this study, have described the capability of H9N2 viruses to adapt to mammals [23,24,30,33,37,38,39], raising concern about their pandemic potential. Even though H9N2 avian viruses have not yet been isolated in domestic poultry in Japan, enhanced surveillance at the border is essential to prevent their spread.

## Figures and Tables

**Table 1:** Molecular markers associated with the adaptation of avian influenza viruses to mammalian hosts found in the viral proteins of the H9N2 viruses Ck/HE28-50 and Ck/HE28-57 ^a^.

Protein	Amino Acid				Viruses Tested in This Study		Phenotype	Subtypes Tested
	Residue		Avian- like Motif	Mammalian-like Motif	Ck/HE28-50	Ck/HE28-57		
PB2	292		I	V	I	V	Increased polymerase activity in mammalian and avian cell lines, increased virulence in mice	H9N2
							Increased polymerase activity in mammalian cell line	H10N8
	504		I	V	V	V	Increased virulence in mice	H9
	588		A	V	V	V	Increased polymerase activity and replication in mammalian and avian cell lines, increased virulence in mice	H7N9, H9N2, H10N8
	89, 309	89	L	V	V	V	Increased polymerase activity and replication in mammalian cells, increased virulence in mice	H5N1
		309	G	D	D	D		
	340,588	340	R	K	R	K	Transmission in guinea pigs	H9N2
		588	A	V	V	V		
PB1	622		D	G	G	G	Increased polymerase activity and enhanced replication in mammalian cell line, increased virulence in mice	H5N1
PA	63		V	I	I	I	Increased polymerase activity and enhanced replication in mammalian cell line, increased virulence in mice	H7N7
	356		K	R	R	R	Increased polymerase activity and replication in mammalian cell line, increased virulence in mice	H9N2
	383		N	D	D	D	Increased polymerase activity in avian and mammalian cell lines	H5N1
HA	159		S	N	N	N	Increased virus binding to α2-6SA	H5N1
	160		T	A	T	A	Increased virus binding to α2-6SA, increased transmission ability in guinea pigs	H5N1
	190		T	V	V	V	Enhanced binding affinity to mammalian cells and replication in mammalian cells	H9N2
	198		N	T	T	T	Increase replication and transmission in ferrets	H9
	226		Q	L	L	L	Increased virus binding to α2-6SA, enhanced replication in mammalian cells and ferrets, enhanced contact transmission in ferrets	H9N2
M1	30		N	D	D	D	Increased virulence in mice	H5N1
	43		I	M	M	M	Increased virulence in mice, chickens, and ducks	H5N1
	215		T	A	A	A	Increased virulence in mice	H5N1
NS	42		P	S	S	S	Increased virulence in mice	H5N1
	138		C	F	F	F	Increased replication mammalian cells, decreased interferon response	H5N1
	149		V	A	A	A	Increased virulence and decreased interferon response	H5N1

^a^, Two H9N2 viruses (Ck/HE28-50 and Ck/HE28-57) possess molecular markers in their viral proteins that are associated with the adaptation of avian influenza viruses to mammalian hosts. A comprehensive list is provided in Supplementary Table S1, with references. HA residue positions are presented with H3 HA numbering.

## Supplementary Materials

The following are available online at https://www.mdpi.com/article/10.3390/v14040728/s1, Table S1: Molecular markers in viral proteins associated with adaptation of avian influenza viruses to mammalian hosts.
