# Supplementary material for: Characterization of H9N2 Avian Influenza Viruses Isolated from Poultry Products in a Mouse Model
Source: Viruses. 2022 Mar 30;14(4):728. doi: 10.3390/v14040728 (PMC9032349; doi:10.3390/v14040728)
Supplement: Supplementary file 1 [file viruses-14-00728-s001.zip › Murakami_Viruses_H9N2mice_Sup Info-rev.pdf]

Supplementary Information

- Supplementary Table S1. Molecular markers in viral proteins associated with adaptation of avian influenza viruses to mammalian hosts. (Excel file)

References in Supplementary Table S1

1. Graef KM, Vreede FT, Lau Y-F, McCall AW, Carr SM, Subbarao K, et al. The PB2 Subunit of the Influenza Virus RNA Polymerase Affects Virulence by Interacting with the Mitochondrial Antiviral Signaling Protein and Inhibiting Expression of Beta Interferon. J Virol. 2010 Sep;84(17):8433–45.

2. Kim JH, Hatta M, Watanabe S, Neumann G, Watanabe T, Kawaoka Y. Role of host-specific amino acids in the pathogenicity of avian H5N1 influenza viruses in mice. J Gen Virol. 2010 May 1;91(5):1284–9.

3. Zhao D, Fukuyama S, Yamada S, Lopes TJS, Maemura T, Katsura H, et al. Molecular Determinants of Virulence and Stability of a Reporter-Expressing H5N1 Influenza A Virus. Dermody TS, editor. J Virol. 2015 Nov 15;89(22):11337–46.

4. Wang J, Sun Y, Xu Q, Tan Y, Pu J, Yang H, et al. Mouse-Adapted H9N2 Influenza A Virus PB2 Protein M147L and E627K Mutations Are Critical for High Virulence. Baker ML, editor. PLoS ONE. 2012 Jul 10;7(7):e40752.

5. Zhou B, Li Y, Halpin R, Hine E, Spiro DJ, Wentworth DE. PB2 Residue 158 Is a Pathogenic Determinant of Pandemic H1N1 and H5 Influenza A Viruses in Mice. J Virol. 2011 Jan;85(1):357–65.

6. Xu G, Wang F, Li Q, Bing G, Xie S, Sun S, et al. Mutations in PB2 and HA enhanced pathogenicity of H4N6 avian influenza virus in mice. J Gen Virol. 2020 Sep 1;101(9):910–20.

7. Taft AS, Ozawa M, Fitch A, Depasse JV, Halfmann PJ, Hill-Batorski L, et al. Identification of mammalian-adapting mutations in the polymerase complex of an avian H5N1 influenza virus. Nat Commun. 2015 Nov;6(1):7491.

8. Zhang J, Su R, Jian X, An H, Jiang R, Mok CKP. The D253N Mutation in the Polymerase Basic 2 Gene in Avian Influenza (H9N2) Virus Contributes to the Pathogenesis of the Virus in Mammalian Hosts. Virol Sin. 2018 Dec;33(6):531–7.

9. Manzoor R, Sakoda Y, Nomura N, Tsuda Y, Ozaki H, Okamatsu M, et al. PB2 Protein of a Highly Pathogenic Avian Influenza Virus Strain A/chicken/Yamaguchi/7/2004 (H5N1) Determines Its Replication Potential in Pigs. J Virol. 2009 Feb 15;83(4):1572–8.

10. Bussey KA, Bousse TL, Desmet EA, Kim B, Takimoto T. PB2 Residue 271 Plays a Key Role in Enhanced Polymerase Activity of Influenza A Viruses in Mammalian Host Cells. J Virol. 2010 May;84(9):4395–406.

11. Foeglein Á, Loucaides EM, Mura M, Wise HM, Barclay WS, Digard P. Influence of PB2 host-range determinants on the intranuclear mobility of the influenza A virus polymerase. J Gen Virol. 2011 Jul 1;92(7):1650–61.

12. Mok CKP, Lee HHY, Lestra M, Nicholls JM, Chan MCW, Sia SF, et al. Amino Acid Substitutions in Polymerase Basic Protein 2 Gene Contribute to the Pathogenicity of the Novel A/H7N9 Influenza Virus in Mammalian Hosts. García-Sastre A, editor. J Virol. 2014 Mar 15;88(6):3568–76.

13. Gao W, Zu Z, Liu J, Song J, Wang X, Wang C, et al. Prevailing I292V PB2 mutation in avian influenza H9N2 virus increases viral polymerase function and attenuates IFN-β induction in human cells. J Gen Virol. 2019 Sep 1;100(9):1273–81.

14. Xiao C, Ma W, Sun N, Huang L, Li Y, Zeng Z, et al. PB2-588 V promotes the mammalian adaptation of H10N8, H7N9 and H9N2 avian influenza viruses. Sci Rep. 2016 May;6(1):19474.

15. Hu M, Yuan S, Zhang K, Singh K, Ma Q, Zhou J, et al. PB2 substitutions V598T/I increase the virulence of H7N9 influenza A virus in mammals. Virology. 2017 Jan;501:92–101.

16. Zhong L, Wang X, Li Q, Liu D, Chen H, Zhao M, et al. Molecular Mechanism of the Airborne Transmissibility of H9N2 Avian Influenza A Viruses in Chickens. J Virol. 2014 Sep 1;88(17):9568–78.

17. Yamayoshi S, Yamada S, Fukuyama S, Murakami S, Zhao D, Uraki R, et al. Virulence-Affecting Amino Acid Changes in the PA Protein of H7N9 Influenza A Viruses. Dermody TS, editor. J Virol. 2014 Mar 15;88(6):3127–34.

18. Yamayoshi S, Kiso M, Yasuhara A, Ito M, Shu Y, Kawaoka Y. Enhanced Replication of Highly Pathogenic Influenza A(H7N9) Virus in Humans. Emerg Infect Dis. 2018 Apr;24(4):746–50.

19. Kandeil A, El-Shesheny R, Maatouq A, Moatasim Y, Cai Z, McKenzie P, et al. Novel reassortant H9N2 viruses in pigeons and evidence for antigenic diversity of H9N2 viruses isolated from quails in Egypt. J Gen Virol. 2017 Apr 1;98(4):548–62.

20. Song W, Wang P, Mok BW-Y, Lau S-Y, Huang X, Wu W-L, et al. The K526R substitution in viral protein PB2 enhances the effects of E627K on influenza virus replication. Nat Commun. 2014 Dec;5(1):5509.

21. Chen G-W, Kuo S-M, Yang S-L, Gong Y-N, Hsiao M-R, Liu Y-C, et al. Genomic Signatures for Avian H7N9 Viruses Adapting to Humans. Davis T, editor. PLOS ONE. 2016 Feb 4;11(2):e0148432.

22. Yamada S, Hatta M, Staker BL, Watanabe S, Imai M, Shinya K, et al. Biological and Structural Characterization of a Host-Adapting Amino Acid in Influenza Virus. Perez DR, editor. PLoS Pathog. 2010 Aug 5;6(8):e1001034.

23. Wang C, Lee HHY, Yang ZF, Mok CKP, Zhang Z. PB2-Q591K Mutation Determines the Pathogenicity of Avian H9N2 Influenza Viruses for Mammalian Species. Pöhlmann S, editor. PLOS ONE. 2016 Sep 29;11(9):e0162163.

24. Kaverin NV, Rudneva IA, Ilyushina NA, Lipatov AS, Krauss S, Webster RG. Structural Differences among Hemagglutinins of Influenza A Virus Subtypes Are Reflected in Their Antigenic Architecture: Analysis of H9 Escape Mutants. J Virol. 2004 Jan 1;78(1):240–9.

25. Hatta M, Hatta Y, Kim JH, Watanabe S, Shinya K, Nguyen T, et al. Growth of H5N1 Influenza A Viruses in the Upper Respiratory Tracts of Mice. Holmes EC, editor. PLoS Pathog. 2007 Oct 5;3(10):e133.

26. Hualan C. Polygenic virulence factors involved in pathogenesis of 1997 Hong Kong H5N1 influenza viruses in mice. Virus Res. 2007;

27. Hatta M. Molecular Basis for High Virulence of Hong Kong H5N1 Influenza A Viruses. Science. 2001 Sep 7;293(5536):1840–2.

28. Shinya K, Hamm S, Hatta M, Ito H, Ito T, Kawaoka Y. PB2 amino acid at position 627 affects replicative efficiency, but not cell tropism, of Hong Kong H5N1 influenza A viruses in mice. Virology. 2004 Mar;320(2):258–66.

29. Fornek JL, Gillim-Ross L, Santos C, Carter V, Ward JM, Cheng LI, et al. A Single-Amino-Acid Substitution in a Polymerase Protein of an H5N1 Influenza Virus Is Associated with Systemic Infection and Impaired T-Cell Activation in Mice. J Virol. 2009 Nov;83(21):11102–15.

30. Le QM, Sakai-Tagawa Y, Ozawa M, Ito M, Kawaoka Y. Selection of H5N1 Influenza Virus PB2 during Replication in Humans. J Virol. 2009 May 15;83(10):5278–81.

31. Mase M, Tanimura N, Imada T, Okamatsu M, Tsukamoto K, Yamaguchi S. Recent H5N1 avian Influenza A virus increases rapidly in virulence to mice after a single passage in mice. J Gen Virol. 2006 Dec 1;87(12):3655–9.

32. Bortz E, Westera L, Maamary J, Steel J, Albrecht RA, Manicassamy B, et al. Host- and Strain-Specific Regulation of Influenza Virus Polymerase Activity by Interacting Cellular Proteins. Katze MG, Virgin H, editors. mBio [Internet]. 2011 Sep [cited 2021 Nov 20];2(4). Available from: https://journals.asm.org/doi/10.1128/mBio.00151-11

33. Bogs J, Kalthoff D, Veits J, Pavlova S, Schwemmle M, Manz B, et al. Reversion of PB2-627E to -627K during Replication of an H5N1 Clade 2.2 Virus in Mammalian Hosts Depends on the Origin of the Nucleoprotein. J Virol. 2011 Oct 15;85(20):10691–8.

34. Airborne Transmission of Influenza A/H5N1 Virus Between Ferrets.

35. PB2-E627K and PA-T97I substitutions enhance polymerase activity and confer a virulent phenotype to an H6N1 avian influenza virus in mice.

36. de Jong RM, Stockhofe-Zurwieden N, Verheij ES, de Boer-Luijtze EA, Ruiter SJ, de Leeuw OS, et al. Rapid emergence of a virulent PB2 E627K variant during adaptation of highly pathogenic avian influenza H7N7 virus to mice. Virol J. 2013 Dec;10(1):276.

37. Zhang H, Li X, Guo J, Li L, Chang C, Li Y, et al. The PB2 E627K mutation contributes to the high polymerase activity and enhanced replication of H7N9 influenza virus. J Gen Virol. 2014 Apr 1;95(4):779–86.

38. Sediri H, Thiele S, Schwalm F, Gabriel G, Klenk H-D. PB2 subunit of avian influenza virus subtype H9N2: a pandemic risk factor. J Gen Virol. 2016 Jan 1;97(1):39–48.

39. Yu Z, Ren Z, Zhao Y, Cheng K, Sun W, Zhang X, et al. PB2 and hemagglutinin mutations confer a virulent phenotype on an H1N2 avian influenza virus in mice. Arch Virol. 2019 Aug;164(8):2023–9.

40. Gao Y, Zhang Y, Shinya K, Deng G, Jiang Y, Li Z, et al. Identification of Amino Acids in HA and PB2 Critical for the Transmission of H5N1 Avian Influenza Viruses in a Mammalian Host. Sherry B, editor. PLoS Pathog. 2009 Dec 24;5(12):e1000709.

41. Steel J, Lowen AC, Mubareka S, Palese P. Transmission of Influenza Virus in a Mammalian Host Is Increased by PB2 Amino Acids 627K or 627E/701N. Baric RS, editor. PLoS Pathog. 2009 Jan 2;5(1):e1000252.

42. Li Z, Chen H, Jiao P, Deng G, Tian G, Li Y, et al. Molecular Basis of Replication of Duck H5N1 Influenza Viruses in a Mammalian Mouse Model. J Virol. 2005 Sep 15;79(18):12058–64.

43. Gabriel G, Dauber B, Wolff T, Planz O, Klenk H-D, Stech J. The viral polymerase mediates adaptation of an avian influenza virus to a mammalian host. Proc Natl Acad Sci. 2005 Dec 20;102(51):18590–5.

44. Gabriel G, Abram M, Keiner B, Wagner R, Klenk H-D, Stech J. Differential Polymerase Activity in Avian and Mammalian Cells Determines Host Range of Influenza Virus. J Virol. 2007 Sep;81(17):9601–4.

45. Li J, Ishaq M, Prudence M, Xi X, Hu T, Liu Q, et al. Single mutation at the amino acid position 627 of PB2 that leads to increased virulence of an H5N1 avian influenza virus during adaptation in mice can be compensated by multiple mutations at other sites of PB2. Virus Res. 2009 Sep;144(1–2):123–9.

46. Fan S, Hatta M, Kim JH, Halfmann P, Imai M, Macken CA, et al. Novel residues in avian influenza virus PB2 protein affect virulence in mammalian hosts. Nat Commun. 2014 Dec;5(1):5021.

47. Lina L, Saijuan C, Chengyu W, Yuefeng L, Shishan D, Ligong C, et al. Adaptive amino acid substitutions enable transmission of an H9N2 avian influenza virus in guinea pigs. Sci Rep. 2019 Dec;9(1):19734.

48. Salomon R, Franks J, Govorkova EA, Ilyushina NA, Yen H-L, Hulse-Post DJ, et al. The polymerase complex genes contribute to the high virulence of the human H5N1 influenza virus isolate A/Vietnam/1203/04. J Exp Med. 2006 Mar 20;203(3):689–97.

49. Zhu W, Zou X, Zhou J, Tang J, Shu Y. Residues 41V and/or 210D in the NP protein enhance polymerase activities and potential replication of novel influenza (H7N9) viruses at low temperature. Virol J. 2015 Dec;12(1):71.

50. Czudai-Matwich V, Otte A, Matrosovich M, Gabriel G, Klenk H-D. PB2 Mutations D701N and S714R Promote Adaptation of an Influenza H5N1 Virus to a Mammalian Host. Sandri-Goldin RM, editor. J Virol. 2014 Aug 15;88(16):8735–42.

51. Linster M, van Boheemen S, de Graaf M, Schrauwen EJA, Lexmond P, Mänz B, et al. Identification, Characterization, and Natural Selection of Mutations Driving Airborne Transmission of A/H5N1 Virus. Cell. 2014 Apr;157(2):329–39.

52. Elgendy EM, Arai Y, Kawashita N, Daidoji T, Takagi T, Ibrahim MS, et al. Identification of polymerase gene mutations that affect viral replication in H5N1 influenza viruses isolated from pigeons. J Gen Virol. 2017 Jan 1;98(1):6–17.

53. Fang S, Wang X, Dong F, Jin T, Liu G, Lu X, et al. Genomic characterization of influenza A (H7N9) viruses isolated in Shenzhen, Southern China, during the second epidemic wave. Arch Virol. 2016 Aug;161(8):2117–32.

54. Kamiki H, Matsugo H, Kobayashi T, Ishida H, Takenaka-Uema A, Murakami S, et al. A PB1-K577E Mutation in H9N2 Influenza Virus Increases Polymerase Activity and Pathogenicity in Mice. Viruses. 2018 Nov 19;10(11):653.

55. Feng X, Wang Z, Shi J, Deng G, Kong H, Tao S, et al. Glycine at Position 622 in PB1 Contributes to the Virulence of H5N1 Avian Influenza Virus in Mice. Dermody TS, editor. J Virol. 2016 Feb 15;90(4):1872–9.

56. Li J, Dohna H zu, Cardona CJ, Miller J, Carpenter TE. Emergence and Genetic Variation of Neuraminidase Stalk Deletions in Avian Influenza Viruses. Boni MF, editor. PLoS ONE. 2011 Feb 23;6(2):e14722.

57. Hu M, Chu H, Zhang K, Singh K, Li C, Yuan S, et al. Amino acid substitutions V63I or A37S/I61T/V63I/V100A in the PA N-terminal domain increase the virulence of H7N7 influenza A virus. Sci Rep. 2016 Dec;6(1):37800.

58. Hu M, Yuan S, Ye Z-W, Singh K, Li C, Shuai H, et al. PA N substitutions A37S, A37S/I61T and A37S/V63I attenuate the replication of H7N7 influenza A virus by impairing the polymerase and endonuclease activities. J Gen Virol. 2017 Mar 1;98(3):364–73.

59. Song M-S, Pascua PNQ, Lee JH, Baek YH, Lee O-J, Kim C-J, et al. The Polymerase Acidic Protein Gene of Influenza A Virus Contributes to Pathogenicity in a Mouse Model. J Virol. 2009 Dec;83(23):12325–35.

60. Kim JH, Hatta M, Watanabe S, Neumann G, Watanabe T, Kawaoka Y. Role of host-specific amino acids in the pathogenicity of avian H5N1 influenza viruses in mice. J Gen Virol. 2010 May 1;91(5):1284–9.

61. Brittany L DesRochers. Residues in the PB2 and PA genes contribute to the pathogenicity of avian H7N3 influenza A virus in DBA/2 mice. Virology.

62. Xu G, Zhang X, Gao W, Wang C, Wang J, Sun H, et al. Prevailing PA Mutation K356R in Avian Influenza H9N2 Virus Increases Mammalian Replication and Pathogenicity. Lyles DS, editor. J Virol. 2016 Sep 15;90(18):8105–14.

63. Song J, Xu J, Shi J, Li Y, Chen H. Synergistic Effect of S224P and N383D Substitutions in the PA of H5N1 Avian Influenza Virus Contributes to Mammalian Adaptation. Sci Rep. 2015 Sep;5(1):10510.

64. Song J, Feng H, Xu J, Zhao D, Shi J, Li Y, et al. The PA Protein Directly Contributes to the Virulence of H5N1 Avian Influenza Viruses in Domestic Ducks. J Virol. 2011 Mar 1;85(5):2180–8.

65. Zhong G, Le MQ, Lopes TJS, Halfmann P, Hatta M, Fan S, et al. Mutations in the PA Protein of Avian H5N1 Influenza Viruses Affect Polymerase Activity and Mouse Virulence. Schultz-Cherry S, editor. J Virol [Internet]. 2018 Feb 15 [cited 2021 Nov 20];92(4). Available from: https://journals.asm.org/doi/10.1128/JVI.01557-17

66. Liang L, Jiang L, Li J, Zhao Q, Wang J, He X, et al. Low Polymerase Activity Attributed to PA Drives the Acquisition of the PB2 E627K Mutation of H7N9 Avian Influenza Virus in Mammals. Dermody TS, editor. mBio [Internet]. 2019 Jun 25 [cited 2021 Nov 20];10(3). Available from: https://journals.asm.org/doi/10.1128/mBio.01162-19

67. Yamaji R, Yamada S, Le MQ, Ito M, Sakai-Tagawa Y, Kawaoka Y. Mammalian Adaptive Mutations of the PA Protein of Highly Pathogenic Avian H5N1 Influenza Virus. Dermody TS, editor. J Virol. 2015 Apr 15;89(8):4117–25.

68. Leung BW, Chen H, Brownlee GG. Correlation between polymerase activity and pathogenicity in two duck H5N1 influenza viruses suggests that the polymerase contributes to pathogenicity. Virology. 2010 May;401(1):96–106.

69. Su Y, Yang H-Y, Zhang B-J, Jia H-L, Tien P. Analysis of a point mutation in H5N1 avian influenza virus hemagglutinin in relation to virus entry into live mammalian cells. Arch Virol. 2008 Dec;153(12):2253–61.

70. Wang W, Lu B, Zhou H, Suguitan AL, Cheng X, Subbarao K, et al. Glycosylation at 158N of the Hemagglutinin Protein and Receptor Binding Specificity Synergistically Affect the Antigenicity and Immunogenicity of a Live Attenuated H5N1 A/Vietnam/1203/2004 Vaccine Virus in Ferrets. J Virol. 2010 Jul;84(13):6570–7.

71. Yamada S, Suzuki Y, Suzuki T, Le MQ, Nidom CA, Sakai-Tagawa Y, et al. Haemagglutinin mutations responsible for the binding of H5N1 influenza A viruses to human-type receptors. Nature. 2006 Nov;444(7117):378–82.

72. Park KJ, Song M-S, Kim E-H, Kwon H, Baek YH, Choi E, et al. Molecular characterization of mammalian-adapted Korean-type avian H9N2 virus and evaluation of its virulence in mice. J Microbiol. 2015 Aug;53(8):570–7.

73. Lu X, Qi J, Shi Y, Wang M, Smith DF, Heimburg-Molinaro J, et al. Structure and Receptor Binding Specificity of Hemagglutinin H13 from Avian Influenza A Virus H13N6. J Virol. 2013 Aug 15;87(16):9077–85.

74. Chen L-M, Blixt O, Stevens J, Lipatov AS, Davis CT, Collins BE, et al. In vitro evolution of H5N1 avian influenza virus toward human-type receptor specificity. Virology. 2012 Jan;422(1):105–13.

75. Sorrell EM, Wan H, Araya Y, Song H, Perez DR. Minimal molecular constraints for respiratory droplet transmission of an avian-human H9N2 influenza A virus. Proc Natl Acad Sci. 2009 May 5;106(18):7565–70.

76. Teng Q, Xu D, Shen W, Liu Q, Rong G, Li X, et al. A Single Mutation at Position 190 in Hemagglutinin Enhances Binding Affinity for Human Type Sialic Acid Receptor and Replication of H9N2 Avian Influenza Virus in Mice. Schultz-Cherry S, editor. J Virol. 2016 Nov 1;90(21):9806–25.

77. Wei Y. Increased virulence of a PB2/HA mutant of an avian H9N2 influenza strain after three passages in porcine differentiated airway epithelial cells. Vet Microbiol. 2017;

78. Yang W, Punyadarsaniya D, Lambertz RLO, Lee DCC, Liang CH, Höper D, et al. Mutations during the Adaptation of H9N2 Avian Influenza Virus to the Respiratory Epithelium of Pigs Enhance Sialic Acid Binding Activity and Virulence in Mice. Lyles DS, editor. J Virol [Internet]. 2017 Apr 15 [cited 2021 Nov 20];91(8). Available from: https://journals.asm.org/doi/10.1128/JVI.02125-16

79. Peng W, Bouwman KM, McBride R, Grant OC, Woods RJ, Verheije MH, et al. Enhanced Human-Type Receptor Binding by Ferret-Transmissible H5N1 with a K193T Mutation. García-Sastre A, editor. J Virol [Internet]. 2018 May 15 [cited 2021 Nov 20];92(10). Available from: https://journals.asm.org/doi/10.1128/JVI.02016-17

80. Watanabe Y, Ibrahim MS, Ellakany HF, Kawashita N, Mizuike R, Hiramatsu H, et al. Acquisition of Human-Type Receptor Binding Specificity by New H5N1 Influenza Virus Sublineages during Their Emergence in Birds in Egypt. Fouchier RAM, editor. PLoS Pathog. 2011 May 26;7(5):e1002068.

81. Lloren KKS, Lee T, Kwon JJ, Song M-S. Molecular Markers for Interspecies Transmission of Avian Influenza Viruses in Mammalian Hosts. Int J Mol Sci. 2017 Dec 13;18(12):2706.

82. Vries RP, Tzarum N, Peng W, Thompson AJ, Ambepitiya Wickramasinghe IN, Pena ATT, et al. A single mutation in Taiwanese H6N1 influenza hemagglutinin switches binding to human-type receptors. EMBO Mol Med. 2017 Sep;9(9):1314–25.

83. Wan H, Perez DR. Amino Acid 226 in the Hemagglutinin of H9N2 Influenza Viruses Determines Cell Tropism and Replication in Human Airway Epithelial Cells. J Virol. 2007 May 15;81(10):5181–91.

84. Wan H, Sorrell EM, Song H, Hossain MJ, Ramirez-Nieto G, Monne I, et al. Replication and Transmission of H9N2 Influenza Viruses in Ferrets: Evaluation of Pandemic Potential. Baylis M, editor. PLoS ONE. 2008 Aug 13;3(8):e2923.

85. Sang X, Wang A, Ding J, Kong H, Gao X, Li L, et al. Adaptation of H9N2 AIV in guinea pigs enables efficient transmission by direct contact and inefficient transmission by respiratory droplets. Sci Rep. 2015 Dec;5(1):15928.

86. Abdelwhab E-SM, Veits J, Breithaupt A, Gohrbandt S, Ziller M, Teifke JP, et al. Prevalence of the C-terminal truncations of NS1 in avian influenza A viruses and effect on virulence and replication of a highly pathogenic H7N1 virus in chickens. Virulence. 2016 Jul 3;7(5):546–57.

87. Reed ML, Yen H-L, DuBois RM, Bridges OA, Salomon R, Webster RG, et al. Amino Acid Residues in the Fusion Peptide Pocket Regulate the pH of Activation of the H5N1 Influenza Virus Hemagglutinin Protein. J Virol. 2009 Apr 15;83(8):3568–80.

88. Krenn BM, Egorov A, Romanovskaya-Romanko E, Wolschek M, Nakowitsch S, Ruthsatz T, et al. Single HA2 Mutation Increases the Infectivity and Immunogenicity of a Live Attenuated H5N1 Intranasal Influenza Vaccine Candidate Lacking NS1. Pekosz A, editor. PLoS ONE. 2011 Apr 7;6(4):e18577.

89. Chen L, Wang C, Luo J, Li M, Liu H, Zhao N, et al. Amino Acid Substitution K470R in the Nucleoprotein Increases the Virulence of H5N1 Influenza A Virus in Mammals. Front Microbiol. 2017 Jul 11;8:1308.

90. Gabriel G, Dauber B, Wolff T, Planz O, Klenk H-D, Stech J. The viral polymerase mediates adaptation of an avian influenza virus to a mammalian host. Proc Natl Acad Sci. 2005 Dec 20;102(51):18590–5.

91. Gabriel G, Herwig A, Klenk H-D. Interaction of Polymerase Subunit PB2 and NP with Importin α1 Is a Determinant of Host Range of Influenza A Virus. Kawaoka Y, editor. PLoS Pathog. 2008 Feb 1;4(2):e11.

92. Li J, Dohna H zu, Cardona CJ, Miller J, Carpenter TE. Emergence and Genetic Variation of Neuraminidase Stalk Deletions in Avian Influenza Viruses. Boni MF, editor. PLoS ONE. 2011 Feb 23;6(2):e14722.

93. Matsuoka Y, Swayne DE, Thomas C, Rameix-Welti M-A, Naffakh N, Warnes C, et al. Neuraminidase Stalk Length and Additional Glycosylation of the Hemagglutinin Influence the Virulence of Influenza H5N1 Viruses for Mice. J Virol. 2009 May;83(9):4704–8.

94. Zhou H, Yu Z, Hu Y, Tu J, Zou W, Peng Y, et al. The Special Neuraminidase Stalk-Motif Responsible for Increased Virulence and Pathogenesis of H5N1 Influenza A Virus. Martin DP, editor. PLoS ONE. 2009 Jul 17;4(7):e6277.

95. Bi Y, Xiao H, Chen Q, Wu Y, Fu L, Quan C, et al. Changes in the Length of the Neuraminidase Stalk Region Impact H7N9 Virulence in Mice. Lyles DS, editor. J Virol. 2016 Feb 15;90(4):2142–9.

96. Park S, Il Kim J, Lee I, Bae J-Y, Yoo K, Nam M, et al. Adaptive mutations of neuraminidase stalk truncation and deglycosylation confer enhanced pathogenicity of influenza A viruses. Sci Rep. 2017 Dec;7(1):10928.

97. Fan S, Deng G, Song J, Tian G, Suo Y, Jiang Y, et al. Two amino acid residues in the matrix protein M1 contribute to the virulence difference of H5N1 avian influenza viruses in mice. Virology. 2009 Feb;384(1):28–32.

98. Nao N, Kajihara M, Manzoor R, Maruyama J, Yoshida R, Muramatsu M, et al. A Single Amino Acid in the M1 Protein Responsible for the Different Pathogenic Potentials of H5N1 Highly Pathogenic Avian Influenza Virus Strains. Takimoto T, editor. PLOS ONE. 2015 Sep 14;10(9):e0137989.

99. Brown EG, Bailly JE. Genetic analysis of mouse-adapted influenza A virus identifies roles for the NA, PB1, and PB2 genes in virulence. Virus Res. 1999 May;61(1):63–76.

100. Kimble JB, Angel M, Wan H, Sutton TC, Finch C, Perez DR. Alternative Reassortment Events Leading to Transmissible H9N1 Influenza Viruses in the Ferret Model. J Virol. 2014 Jan 1;88(1):66–71.

101. Jiao P, Tian G, Li Y, Deng G, Jiang Y, Liu C, et al. A Single-Amino-Acid Substitution in the NS1 Protein Changes the Pathogenicity of H5N1 Avian Influenza Viruses in Mice. J Virol. 2008 Feb 1;82(3):1146–54.

102. Heui Seo S, Hoffmann E, Webster RG. Lethal H5N1 influenza viruses escape host anti-viral cytokine responses. Nat Med. 2002 Sep;8(9):950–4.

103. Long J-X, Peng D-X, Liu Y-L, Wu Y-T, Liu X-F. Virulence of H5N1 avian influenza virus enhanced by a 15-nucleotide deletion in the viral nonstructural gene. Virus Genes. 2008 Jun;36(3):471–8.

104. Kuo R-L, Krug RM. Influenza A Virus Polymerase Is an Integral Component of the CPSF30-NS1A Protein Complex in Infected Cells. J Virol. 2009 Feb 15;83(4):1611–6.

105. Spesock A, Malur M, Hossain MJ, Chen L-M, Njaa BL, Davis CT, et al. The Virulence of 1997 H5N1 Influenza Viruses in the Mouse Model Is Increased by Correcting a Defect in Their NS1 Proteins. J Virol. 2011 Jul 15;85(14):7048–58.

106. Li J, Zhang K, Chen Q, Zhang X, Sun Y, Bi Y, et al. Three amino acid substitutions in the NS1 protein change the virus replication of H5N1 influenza virus in human cells. Virology. 2018 Jun;519:64–73.

107. Li Z, Jiang Y, Jiao P, Wang A, Zhao F, Tian G, et al. The NS1 Gene Contributes to the Virulence of H5N1 Avian Influenza Viruses. J Virol. 2006 Nov;80(22):11115–23.

108. Zielecki F, Semmler I, Kalthoff D, Voss D, Mauel S, Gruber AD, et al. Virulence Determinants of Avian H5N1 Influenza A Virus in Mammalian and Avian Hosts: Role of the C-Terminal ESEV Motif in the Viral NS1 Protein. J Virol. 2010 Oct 15;84(20):10708–18.

109. Soubies SM, Volmer C, Croville G, Loupias J, Peralta B, Costes P, et al. Species-Specific Contribution of the Four C-Terminal Amino Acids of Influenza A Virus NS1 Protein to Virulence. J Virol. 2010 Jul;84(13):6733–47.

110. Reuther P, Giese S, Gotz V, Kilb N, Manz B, Brunotte L, et al. Adaptive Mutations in the Nuclear Export Protein of Human-Derived H5N1 Strains Facilitate a Polymerase Activity-Enhancing Conformation. J Virol. 2014 Jan 1;88(1):263–71.
